# Inbreeding removes sex differences in lifespan in a population of *Drosophila melanogaster*

**DOI:** 10.1098/rsbl.2016.0337

**Published:** 2016-06

**Authors:** Pau Carazo, Jared Green, Irem Sepil, Tommaso Pizzari, Stuart Wigby

**Affiliations:** 1 Ecology, Ethology and Evolution Group, Cavanilles Institute of Biodiversity and Evolutionary Biology, University of Valencia, C/ Catedrático José Beltrán n°2, CP: 46980, Paterna, Valencia, Spain; 2 Edward Grey Institute, Department of Zoology, University of Oxford, Oxford OX1 3PS, UK

**Keywords:** ageing, unguarded-X, asymmetric inheritance

## Abstract

Sex differences in ageing rates and lifespan are common in nature, and an enduring puzzle for evolutionary biology. One possibility is that sex-specific mortality rates may result from recessive deleterious alleles in ‘unguarded’ heterogametic X or Z sex chromosomes (the unguarded X hypothesis). Empirical evidence for this is, however, limited. Here, we test a fundamental prediction of the unguarded X hypothesis in *Drosophila melanogaster*, namely that inbreeding shortens lifespan more in females (the homogametic sex in *Drosophila*) than in males. To test for additional sex-specific social effects, we studied the lifespan of males and females kept in isolation, in related same-sex groups, and in unrelated same-sex groups. As expected, outbred females outlived outbred males and inbreeding shortened lifespan. However, inbreeding-mediated reductions in lifespan were stronger for females, such that lifespan was similar in inbred females and males. We also show that the social environment, independent of inbreeding, affected male, but not female lifespan. In conjunction with recent studies, the present results suggest that asymmetric inheritance mechanisms may play an important role in the evolution of sex-specific lifespan and that social effects must be considered explicitly when studying these fundamental patterns.

## 1. Introduction

Sex differences in ageing and lifespan are near ubiquitous in nature, yet little is known about the mechanisms causing these patterns. Three main complementary theories could explain sex-specific lifespan. First, sex-specific lifespans may reflect differences in selective pressures and age-dependent risk of extrinsic mortality, such as those associated with sexual selection [1]. Second, sex-specific lifespans might be caused by the asymmetric inheritance of cytoplasmic genomes (mother's curse hypothesis), where selection can only purge mitochondrial mutations in females [2,3]. Finally, the accumulation of recessive deleterious mutations in the X or Z sex chromosomes [4] could generate sex differences in lifespan, because these mutations will be unconditionally expressed in the heterogametic sex, but not in the homogametic sex (the unguarded X hypothesis). Patterns of sex-specific lifespan in vertebrates are broadly consistent with the unguarded X hypothesis [5], but few experimental studies explicitly test predictions arising from the theory, and what few studies are available have produced inconsistent results [6]. A possible reason for these inconsistencies may be that these studies did not control for social context, whose potentially critical role is very often neglected in studies of sex-specific lifespan. Social effects, such as the presence or absence of intraspecific competitors or the degree of relatedness of such competitors, are known to influence behaviour and lifespan in a sex-specific way [7].

Here, we test a fundamental prediction of the unguarded X hypothesis: that the negative effect of inbreeding on lifespan should be more pronounced in the homogametic sex than in the heterogametic sex. This prediction arises because, in the heterogametic sex (e.g. males in XY systems and females in ZW systems), the sex chromosomes are always ‘effectively inbred’, thus only the homogametic sex should suffer additional survival costs from increased homozygosis under inbreeding. We first constructed inbred lines, and conducted crosses to generate outbred, intermediately inbred, and fully inbred flies (see [8,9]). To explore the potential interactions with social effects [8], we measured the lifespan of these flies in the presence or absence of same-sex competitors. Furthermore, we varied the degree of relatedness of the competitors, because this is known to influence behaviour and lifespan in a sex-specific way [10].

## 2. Methods

**Figure 1.**
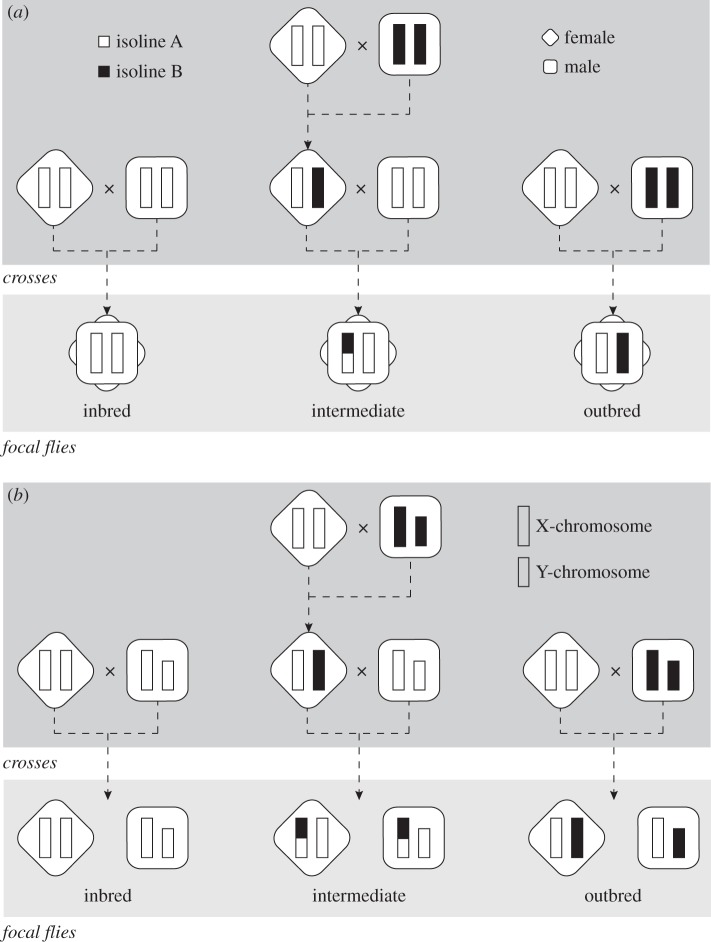
Schematic design of crosses used to construct the three inbreeding treatments (i.e. outbred, intermediately inbred and inbred) from randomly paired isolines (adapted from [8]) and resulting inbreeding levels in: (*a*) autosomes and (*b*) sex chromosomes. Note that there is no meiotic recombination in *D. melanogaster* males.

We used flies from a wild-type Dahomey stock of *D. melanogaster* [11] that has been maintained in population cages with overlapping generations (i.e. permitting full lifespan) at 25°C since 1970. Unrelated flies were paired and 10 generations of consecutive full-sib crosses produced 38 replicate isolines (inbreeding coefficient of at least approx. 0.886; for details, see [12]). We then randomly paired the lines to create 19 ‘sets’. Within each set, we arranged the crosses to produce three inbreeding levels: (i) fully inbred, (ii) outbred and (iii) intermediately inbred (figure 1; adapted from [8]). Virgin flies from these crosses were maintained under three different social environments: (i) individuals in isolation (two replicate vials per set, per sex), (ii) grouped with same-sex relatives (one vial of five flies per set, per sex) and (iii) grouped with same-sex non-relatives (one vial with one focal fly per sex per set, grouped with four rival flies of the same-sex and Dahomey genetic background who possessed a *spa* eye phenotype; [13]). Flies were transferred to fresh vials once a week for the isolated treatment, and twice a week for the group treatments, using light CO_2_ anaesthesia. Vials were checked 5–6 days a week for deaths, and dead *spa* flies were replaced. Lifespan of flies in the grouped with relatives treatment was calculated as the mean lifespan for the five focal flies. For the methods above, we collected Dahomey eggs from population cages on grape-agar-filled Petri dishes, smeared with live yeast paste. The eggs were placed at a standardized density of 10 µl (approx. 180 eggs) per 75 ml bottle containing approximately 45 ml fly food using the protocol described in Clancy & Kennington [14]. Virgin males and females were collected from these bottles within 8 h of eclosion and aged in groups of 15 in vials for 48 h.

We used a restricted maximum-likelihood (LMMs) approach, with sex, social environment, inbreeding level and their interactions (including the three-way interaction) as fixed factors and set as a random intercept effect. Models were checked for assumptions and alpha-winsorized (*α* = 0.05; [15]) to control from outliers. Models were simplified by backward single term deletions (*p* ≤ 0.05) followed by model comparisons via likelihood ratio tests. Significant sex interactions were explored by fitting LMMs separately for both sexes. All analyses were performed in R v. 3.1.2 [16]. Data available in Dryad [17].

## 3. Results

Model selection produced a final model including sex, social environment treatment, inbreeding level, and sex × inbreeding level and sex × social environment treatment interactions as the fixed effects.

### (a) Male–female comparisons

**Figure 2.**
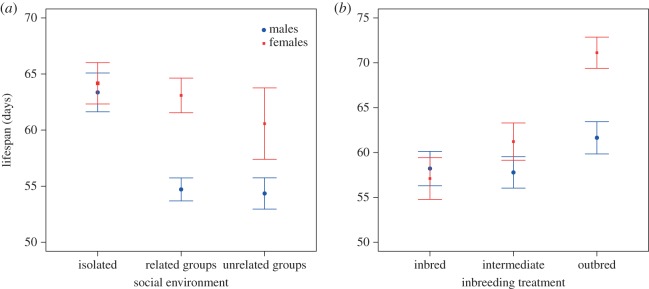
Male and female lifespan (mean ± s.e.) across social environments (*a*) and inbreeding levels (*b*). (Online version in colour.)

The sex-specific patterns described above resulted in significant sex × inbreeding level (d.f. = 2, *χ*^2^ = 8.385, *p* = 0.015) and sex × social environment treatment (d.f. = 2, *χ*^2^ = 10.332, *p* = 0.006) interactions. Male lifespan was more affected by same-sex competitors than female lifespan (figure 2*a*). On the other hand, female lifespan was more affected by inbreeding depression than male lifespan, so that the lifespan of inbred females was as short as that of inbred males even though outbred females outlived outbred males (figure 2*b*).

### (b) Males

There were significant effects of social environment (d.f. = 2, *χ*^2^ = 31.226, *p* < 0.001) and inbreeding (d.f. = 2, *χ*^2^ = 6.287, *p* = 0.043) on male lifespan. *Post hoc* Tukey contrasts indicated that males in isolation had longer lifespans than males grouped with relatives (estimate = 9.149 ± 1.958, *z* = 4.672, *p* < 0.001) and with non-relatives (estimate = 9.547 ± 2.045, *z* = 4.669, *p* < 0.001), whereas the latter two exhibited similar lifespans (estimate = −0.398 ± 2.360, *z* = −0.169, *p* = 0.984). Furthermore, outbred males had significantly longer lifespans than intermediately inbred (estimate = 4.694 ± 1.976, *z* = 2.375, *p* = 0.046), but not inbred (estimate = 3.675 ± 1.970, *z* = 1.866, *p* = 0.149) flies, whereas intermediately inbred and inbred flies exhibited similar lifespans (estimate = −1.019 ± 1.998, *z* = −0.510, *p* = 0.866; table 1 and electronic supplementary material, S1 and figure 2*a*,*b*).

**Table 1.** Parameter estimates for final sex-specific models (see §3).

sex	factor	estimate	s.e.	*t*-value
males	intercept	63.022	1.989	31.69
	inbreeding (intermediate)	−1.019	1.998	−0.51
	inbreeding (outbred)	3.675	1.970	1.87
	social environment (related)	−9.149	1.958	−4.67
	social environment (unrelated)	−9.547	2.045	−4.67
females	intercept	59.384	2.004	29.638
	inbreeding (intermediate)	3.199	2.252	1.421
	inbreeding (outbred)	12.002	2.268	5.292
	social environment (related)	−1.301	2.234	−0.582
	social environment (unrelated)	−1.808	2.312	−0.782

### (c) Females

There were significant effects of inbreeding (d.f. = 2, *χ*^2^ = 27.778, *p* < 0.001) but not social environment (d.f. = 2, *χ*^2^ = 0.759, *p* = 0.684) on female lifespan (table 1 and electronic supplementary material, S1 and figure 2*a*,*b*). *Post hoc* Tukey contrasts indicated that inbred females had shorter lifespans than both outbred females (estimate = −12.002 ± 2.268, *z* = −5.292, *p* < 0.001) and intermediately inbred females (estimate = −8.803 ± 2.268, *z* = −3.881, *p* < 0.001), whereas the latter two exhibited similar lifespans (inbred-intermediate: estimate = −3.199 ± 2.252, *z* = −1.421, *p* = 0.330).

## 4. Discussion

In this study, we report that, in our population of *D. melanogaster*, inbreeding has sex-specific effects on lifespan that are consistent with predictions from the unguarded X hypothesis. We detected a clear sex by inbreeding interaction, whereby inbreeding caused a strong reduction in female lifespan but had a much lesser effect on male lifespan. Hence, inbreeding erased the sexual dimorphism in lifespan typical of *D. melanogaster*, and many other dioecious organisms. Our data further show that sexually dimorphic lifespan was recovered by the rescue effect of outbreeding on female lifespan. Therefore, in contrast with previous studies in seed beetles (*Callosobruchus maculatus*) [8,9], inbreeding negatively affected both female and male lifespan and, as predicted by the unguarded X hypothesis, this effect was larger in the homogametic sex (females in *D. melanogaster*). Although we did not measure reproductive inbreeding depression here, a recent study in our same laboratory population found that inbreeding reduces egg-to-adult viability more in female than in male offspring [18], which is in line with our findings and with predictions from the unguarded X hypothesis (but see [19]). Finally, in our study, male and female autosomes were, on average, subject to the same degree of inbreeding. However, studies that have measured inbreeding have frequently found that one of the sexes is generally more vulnerable than the other over a wide range of fitness-related traits [20]. It is thus also possible that *Drosophila* females are generally more vulnerable to lifespan-related inbreeding depression that males, and this alternative should be considered in future studies.

Our results also confirm that social environment can have sex-specific effects on lifespan, but we detected no interacting effects with inbreeding levels. Previous studies have measured the lifespan of individuals that were either kept isolated in a vial [9] or in same-sex groups [8], but did not experimentally test the social environment as a factor. While measuring the lifespan of individuals in isolation has the advantage of eliminating the effect of social influences, it might not necessarily capture ecologically relevant conditions because in nature, flies will typically engage in social interactions. We implemented a fully factorial inbreeding level by social environment treatment design and found that social environment had no effect on female ageing, but a marked effect on male ageing, whereby males in groups aged more quickly than males in isolation. An interesting avenue for further research would involve looking at inbreeding effects in mixed-sexed contexts under varying levels of sexual conflict. Our results are nevertheless consistent with previous evidence showing that male *D. melanogaster* exhibit higher levels of intrasexual aggression and territoriality than females, and that selection in this species is stronger in males [21]. More generally, they converge with existing evidence to suggest that sexual dimorphism in lifespan is mainly driven by sex-specific adaptive selection [6].

The question of why sexes age differently is an enduring challenge with broad evolutionary implications. There is increasing evidence that sex-specific adaptive evolution is fundamental to understanding the evolution of sex differences in ageing and lifespan [1,6], but the mechanisms underpinning sex-specific lifespan remain unclear. Our results suggest the unguarded X hypothesis could also underlie sex differences in ageing in a *D. melanogaster* population which, along with recent studies (e.g. [5,22,23]), underscores the fact that asymmetric inheritance processes may act in conjunction with sex-specific selection to shape sex-specific lifespan. Asymmetric inheritance processes are particularly alluring as they complement explanations based on sex-specific adaptive processes and can potentially act at a wide taxonomic level, and hence deserve more attention.

## Data accessibility

Data are available from the Dryad digital repository: http://dx.doi.org/10.5061/dryad.pp6k7.
